# Development of a screening algorithm for borderline personality disorder using electronic health records

**DOI:** 10.1038/s41598-022-16160-z

**Published:** 2022-07-13

**Authors:** Chengxi Zang, Marianne Goodman, Zheng Zhu, Lulu Yang, Ziwei Yin, Zsuzsanna Tamas, Vikas Mohan Sharma, Fei Wang, Nan Shao

**Affiliations:** 1 Department of Population Health Sciences, Weill Cornell Medicine, New York, NY USA; 2 Department of Psychiatry, Icahn School of Medicine at Mount Sinai, New York, NY USA; 3 James J Peters VA Medical Center, Bronx, NY USA; 4 Boehringer Ingelheim Pharmaceuticals, Inc. Ridgefield, Ridgefield, CT USA; 5 Boehringer Ingelheim International GmbH, Ingelheim am Rhein, Germany

**Keywords:** Psychiatric disorders, Health care

## Abstract

Borderline personality disorder (BoPD or BPD) is highly prevalent and characterized by reactive moods, impulsivity, behavioral dysregulation, and distorted self-image. Yet the BoPD diagnosis is underutilized and patients with BoPD are frequently misdiagnosed resulting in lost opportunities for appropriate treatment. Automated screening of electronic health records (EHRs) is one potential strategy to help identify possible BoPD patients who are otherwise undiagnosed. We present the development and analytical validation of a BoPD screening algorithm based on routinely collected and structured EHRs. This algorithm integrates rule-based selection and machine learning (ML) in a two-step framework by first selecting potential patients based on the presence of comorbidities and characteristics commonly associated with BoPD, and then predicting whether the patients most likely have BoPD. Leveraging a large-scale US-based de-identified EHR database and our clinical expert’s rating of two random samples of patient EHRs, results show that our screening algorithm has a high consistency with our clinical expert’s ratings, with area under the receiver operating characteristic (AUROC) 0.837 [95% confidence interval (CI) 0.778–0.892], positive predictive value 0.717 (95% CI 0.583–0.836), accuracy 0.820 (95% CI 0.768–0.873), sensitivity 0.541 (95% CI 0.417–0.667) and specificity 0.922 (95% CI 0.880–0.960). Our aim is, to provide an additional resource to facilitate clinical decision making and promote the development of digital medicine.

## Introduction

Borderline personality disorder (BoPD) is a serious psychological condition that affects 1–2% of the general population, and approximately $10\%$ and $20\%$ of psychiatric outpatients and inpatients, respectively^1,2^. BoPD is characterized by chronic disinhibition, extreme sensitivity, volatile emotions, self-harm, impulsive behaviors and high mortality rate due to suicide (up to $10\%$)^3,4^.The diagnosis of BoPD is often overlooked with almost $40\%$ reporting a previous misdiagnosis compared to only $10\%$ of patients with other psychological disorders^5^, and even when identified, many patients do not receive appropriate treatments.

The gold standard for diagnosing BoPD is the Structured Clinical Interview for DSM-5, also known as SCID^6^, which is a lengthy interview often reserved for research settings. BoPD screeners do exist including: (1) the McLean Screening Instrument for Borderline Personality Disorder (MSI-BPD) (10 items^7^), (2) Borderline Personality Questionnaire (80 questions^8^), (3) The Borderline Symptoms List (23 items^9^). These instruments are all self-report, with limited validity due to retrospective memory difficulties especially with respect to mood instability seen in patients with BoPD^10^. An alternative method of screening for mental illness is through examination of provider data in medical records, including historical information from progress notes^11^, and details from initial presentations^12^. However, these efforts have not yet been extended to individuals with BoPD.

Advances in health data and analytics have already led to numerous opportunities to improve care delivery and benefit patients^13–16^. Specifically, machine learning (ML) models building on electronic health records (EHRs) have been used to predict risk of mental health conditions, such as postpartum depression^17^ and suicide in children and adolescents^18^. Examples of real-world implementation include a high-risk monitoring system in the Veterans Administration (VA) Medical centers based on EHR data to stratify suicide risk^19^. We believe that using ML to perform automated screening of EHRs which contain routinely collected longitudinal patient health information is one potentially helpful strategy to address the unmet need for the identification of possible BoPD patients who are mis- or not diagnosed. However, the application of ML in BoPD is limited, let alone real-world implementations. One study^20^ investigated the risk factors for future BoPD symptoms using regularized regression on a prospective longitudinal dataset of adolescent girls, which seems to be the only ML application in BoPD to-date. To the best of our knowledge, our project to build a ML model for BoPD screening based on EHRs is the first of its kind.

The proposed method follows a two-step approach by first selecting potential patients based on rules on the presence of comorbidities and characteristics commonly associated with BoPD when BoPD has not been formally diagnosed, and then narrowing down the recommendation to health care professionals (HCPs) by predicting whether the patients are most likely to have BoPD using ML.

One of major challenges in the ML development for medicine is the huge gap between the scarcity of expert annotated data (referred to as gold-labels) and the overabundance of unlabelled health data majorly due to the costly annotation process by domain experts. The ML component of our screening method followed a semi-supervised learning idea^21–24^ and leveraged a small amount of labeled data with a large amount of unlabeled data. During the development of our ML models, we built a 90 times larger training data set with silver-labels (generated without expert annotation) than the initial gold-label training data, leading to a significant improvement in generalization performance. We also found that rather than self-reinforcing knowledge learned from the gold-label data, introducing additional knowledge in building the silver-label data can improve the model performance, and such additional knowledge should not be as expensive as domain expert annotation.

We leveraged a large-scale US-based de-identified EHR database which includes 13 million adult subjects from 2015 to 2018 for the algorithm development. Our BoPD clinical expert’s opinion on the likelihood of a patient having BoPD was applied to two random samples of patients’ EHRs, providing necessary data for building such screening algorithm. In this paper, we examine the performance of the ML component utilizing the clinical expert’s rating and report the results.

## Results

### Rule-based selection for potential BoPD patients in Cerner

The US Cerner Health Facts (Cerner Corp., Kansas City, MO) database was used for this study. Of the 13,257,125 adult subjects ($\ge$18 years of age) in Cerner who had $\ge 1$ ICD-10-CM diagnosis code during study period (encounter discharge date from October 1, 2015 to July 11,2018), 183,475 (5%) were identified as potential patients with BoPD based on common comorbid conditions or key characteristics associated with the disorder. Additionally, subject inclusion required sufficient medical history in order for the algorithm to predict outcomes. Specifically, 1,1811,105 out of 13,257,125 (14%) subjects were retained initially with presence of mental disorder associated with BoPD, 1,040,704 out of 1,1811,105 (57%) subjects were retained for having sufficient medical history, and finally 183,475 out of 1,040,704 (18%) subjects were retained as potential patients with BoPD if they had suicidal/intentional self-harm or a diagnosis of bipolar disorder, or a history of mental disorders in at least 3 other pre-defined groups during study period. Pull-through rates may differ across other healthcare organizations. Details on selection rule can be found in the Methods section and additional information of potential BoPD cohort in Cerner can be found in Supplementary Table 1.

### ML model development and performance

Our BoPD clinical expert (M.G., former President of North American Society for the Study of Personality Disorders) reviewed 456 patient records from two independent random samples (228 patients in each sample) from the potential BoPD cohort, and clinically rated each patient on the likelihood of having BoPD. Patient records were structured EHR data including routinely collected information such as demographics, diagnosis codes, encounter types and dates. Patients with rating category “Most likely BoPD” are considered as candidates for formal clinical assessment for BoPD and therefore this rating category is the positive label in the binary classification task. Other rating categories including “no potential” or “weak potential” of BoPD were combined as negative labels. The two rated sets used for training and testing were referred to as gold-label training data and gold-label testing data. The percentage of positive cases were 29% (66/228) and 27% (61/228) in gold-label training and gold-label testing data, respectively (proportion test, $p = 0.676$). A subset of positive cases were further labelled as having characteristics associated with classic BoPD (12 and 9 in gold-label training and gold-label testing data, respectively). The algorithm was expected to identify classic BoPD as much as possible and therefore sensitivity of classic BoPD is included as an evaluation metric in addition to other performance metrics.

Following a semi-supervised learning framework, we then built a 90 times larger silver-label training data, including 20, 592 patients records with 5961 (29%) silver-positives and 14,631 (71%) silver-negatives. Silver-positives were identified with a rule-based selection process from a patient cohort already with BoPD diagnosis code, which were referred to as “EHR diagnosed BoPD cohort”. Some patients in EHR diagnosed BoPD cohort were excluded because they had limited diagnostic history in the medical records which precluded our ability to identify relevant comorbidities, which was necessary for building silver-positives. Silver-negatives were selected from predicted negative in the potential BoPD cohort using the logistic regression (LR) model with L-1 norm regularization trained on gold-label data. A detailed description of gold-label and silver-label data can be found in the Methods section. The demographics and encounter types in gold-label and silver-label data sets are summarized in Table 1. The gender and age distributions are identical in two gold-label sets because stratified sampling was applied, and details can be found in the Methods section.

**Table 1:** Demographics and encounter types in gold-label and silver-label data sets.

	Gold-label training	Silver-label training	Gold label training subset	Gold-label testing
No.	228	20,592	94	228
No. (%) of positive cases	66 (28.9%)	5,961 (28.9%)	66 (70.2%)	61 (26.8%)
Age no. (%)				
18-39	104 (45.6%)	9,404 (45.7%)	52 (55.3%)	104 (45.6%)
40-59	104 (45.6%)	9,467 (46.0%)	37 (39.4%)	104 (45.6%)
60-65	20 (8.8%)	1,721 (8.4%)	5 (5.3%)	20 (8.8%)
Female no. (%)	137 (60.1%)	14,297 (69.4%)	43 (45.7%)	137 (60.1%)
Encounter type no. (%)				
Emergency	913 (27.9%)	84,060 (21.9%)	479 (42.1%)	1,039 (30.9%)
Inpatient	202 (6.2%)	22,983 (6.0%)	112 (9.8%)	208 (6.2%)
Outpatient and others	2,152 (65.9%)	276,260 (72.1%)	548 (48.1%)	2,119 (63.0%)

**Figure 1:**
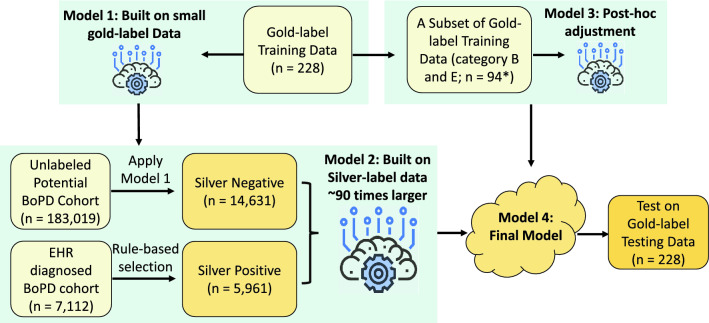
The ML model development pipeline which follows a semi-supervised learning framework by starting with small gold-label data, and then building a large silver-label data; an adjustment model was built to reduce false positives. Details can be found in the Methods section. *Refer to the Table 3 for the label categories.

The main model was built on silver-label training data using LR with L-1 norm regularization. We then built an adjustment model based on a subset of the gold-label data using LR with L1-norm regularization to further distinguish a subset of the negative cases with rating category “severe psychotic/substance abuse” from the positive cases. We combined the predictions from the main model and the adjustment model to reduce false positives. And the combined prediction is the final prediction. The overall ML model development pipeline is illustrated in Fig. 1. More details on the development pipeline including feature engineering can be found in the Methods section.

Performance of the final model was evaluated on gold-label testing data, and the area under the receiver operating characteristics curve (AUROC) is 0.837 (95% CI: 0.778–0.892), and positive predictive value is 0.717 (95% CI: 0.583–0.836) indicating that for every 10 patients identified by the algorithm, on average 7 of them are most likely to be patients with BoPD. Accuracy is 0.820 (95% CI: 0.768–0.873) and sensitivity and specificity are 0.541 (95% CI: 0.417–0.667) and 0.922 (95% CI: 0.880-0.960), respectively. In addition, the recall of classic BoPD is 1.000 (95% CI: 1.000–1.000), demonstrating that the model is capable of capturing all classic BoPD cases.

**Figure 2:**
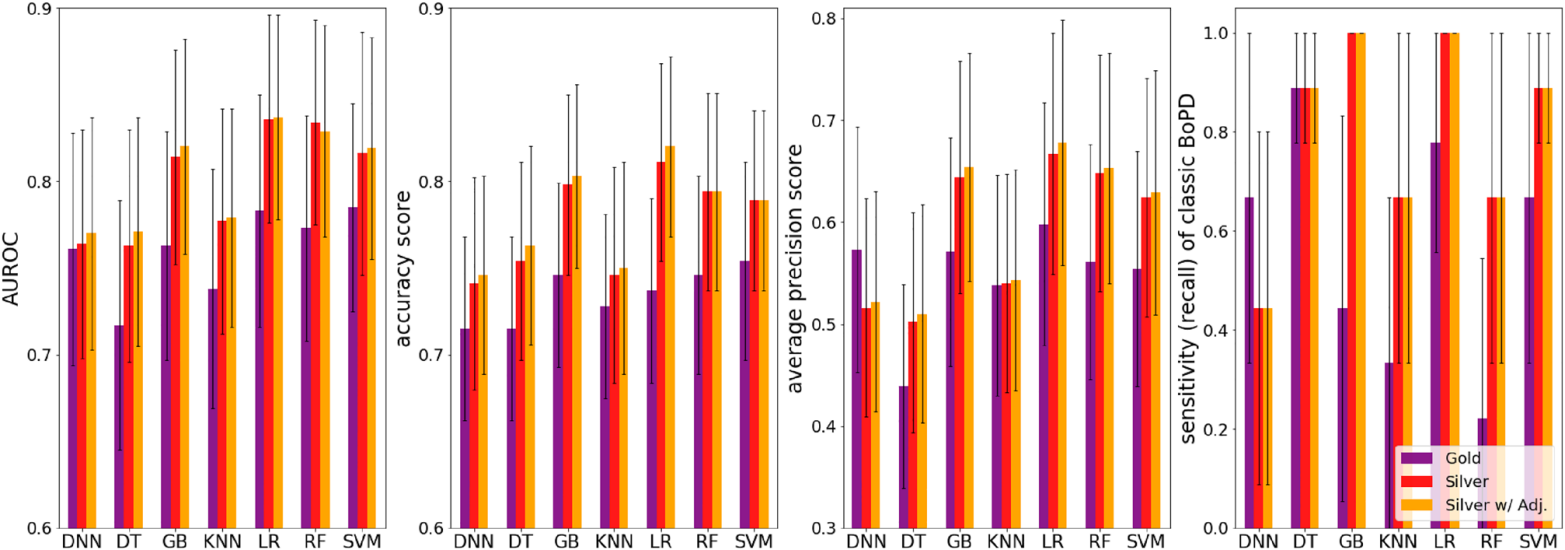
Model performance comparison among models trained on gold-label training data only, silver-label training data, and silver-label training data with the adjustment model. Black verticle lines represent 95% confidence interval. DNN, deep neural network; DT, decision tree; GBM, gradient boosting machine; KNN, K-nearest neighbors; LR, regularized logistic regression; RF, random forest; SVM, supportive vector machine.

**Figure 3:**
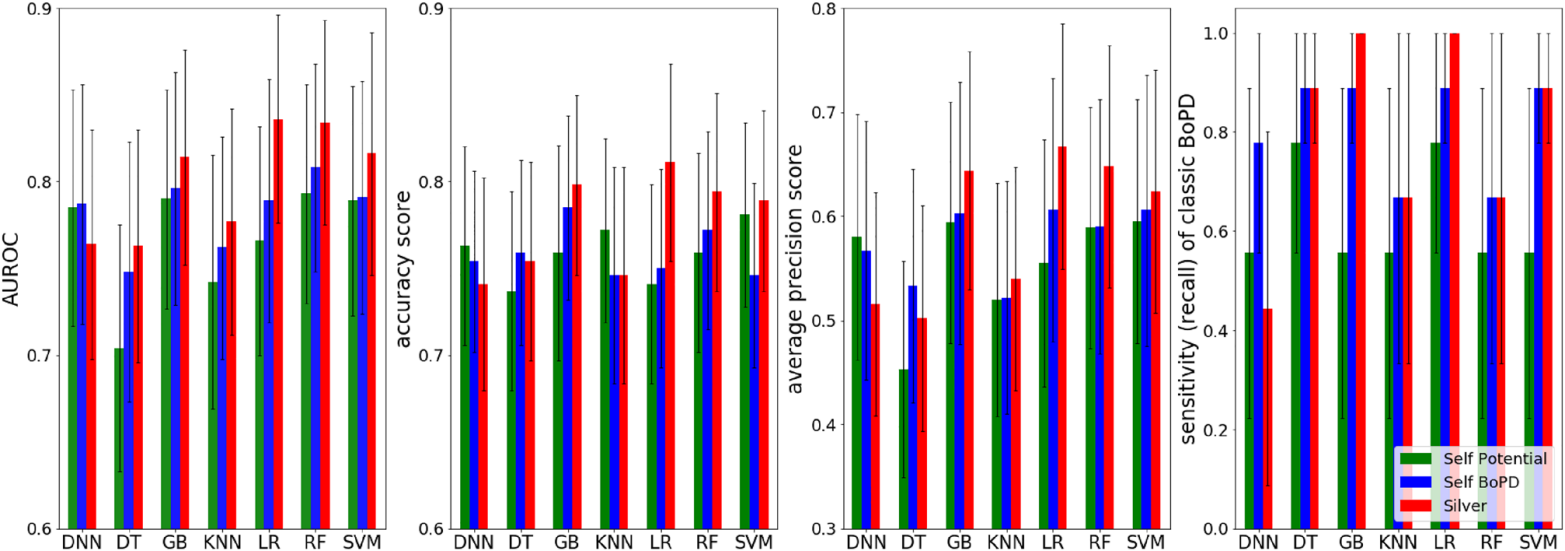
Model performance comparison among models trained on different silver-positives. Self-potential (green bars) and self-BoPD (blue bars): silver-positives were built via applying the LR model trained on gold-label data on potential BoPD cohort and EHR diagnosed BoPD cohort, respectively, to generate predicted positives. Silver (red bars): rule-based selection of silver-positives from EHR diagnosed BoPD cohort, also called knowledge-enriched silver-positives. Black verticle lines represent 95% confidence interval.

We compared performance of LR with other machine learning models trained on different data sets (illustrated in Figs. 2 and 3). Our results indicate that logistic regression out-performed other models, namely, bar groups corresponding to LR in both figures are usually the tallest among other bar groups. The detailed numeric results can be found in Supplementary Table 2.

In addition, to demonstrate the benefits of building silver-label training data and making further adjustments, we compared performance from models trained on gold-label training data only, silver-label training data, and silver-label training data with our adjustment model. In summary, models trained on silver-label data consistently outperform models trained on gold-label data regardless of model types, and the adjustment improves model performance further, as can be observed in Fig. 2. Taking LR for example, the AUROC is 0.783 ($95\%$ CI $0.716-0.849$) for gold-label data, 0.836 ($95\%$ CI $0.776-0.891$) for silver-label data and 0.837 ($95\%$ CI $0.778-0.892$) for silver-label data with adjustment. The sensitivity of classic BoPD is improved from 0.778 (95% CI 0.429–1.000) to 1.000 (95% CI 1.000–1.000).

Moreover, our proposed way of building the silver-positives is rule-based and it brings in knowledge in addition to those learned from the gold-label training data. The model performance based on such “knowledge-enriched” silver-positives is illustrated as red bars in Fig. 3. To demonstrate the benefits, we compared the model performance with two other ways of building silver-positives by only self-reinforcing knowledge learned from the gold-label training data. And these two ways are to apply the LR model trained on gold-label data on potential BoPD cohort and EHR diagnosed BoPD cohort, respectively, to generate predicted positives. The corresponding model performances are illustrated as green and blue bars in Fig. 3. In general, the red bar is taller than the green and blue bars in each bar group in each sub-figure, with a few exceptions such as the DNN model and decision tree model, which are not good performing models anyway.

### Feature analysis for the ML model

**Figure 4:**
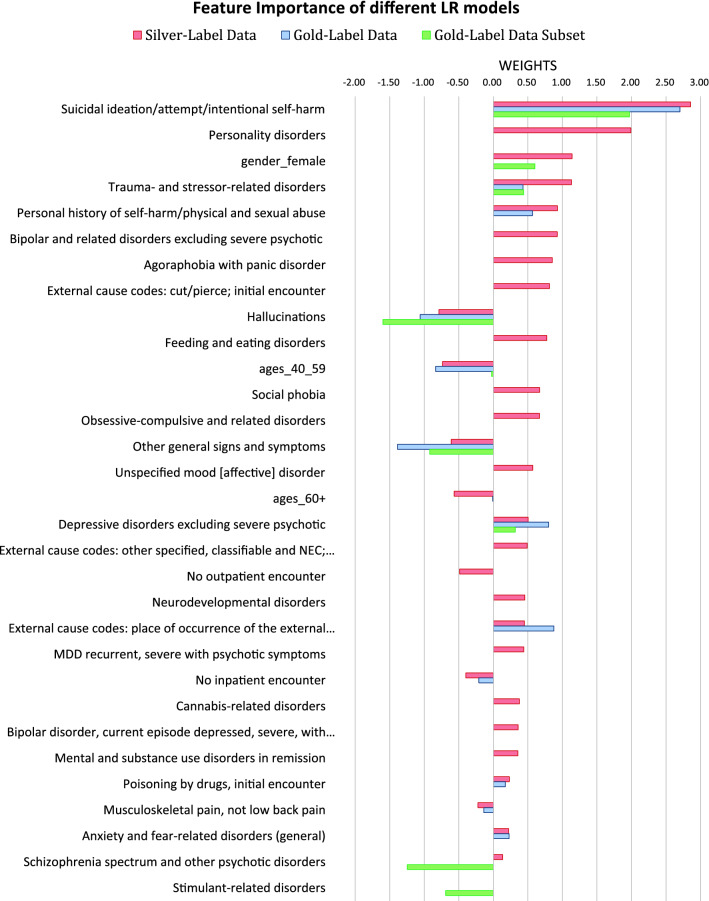
Important features and their weights in the models trained on silver-label data (main model), gold-label data (initial model), and subset of gold-label data (adjustment model). Missing bars represent zero weights, namely no contribution, of corresponding features to the final predictive results.

When examining the important features in the main model (red bars in Fig. 4), we observed that many features, typically associated with clinical presentations of BoPD had large positive weights, such as “suicidal ideation/attempt/intentional self-harm”, “personality disorders”, “trauma and stressor related disorders”, “personal history of self-harm/physical and sexual abuse”, “bipolar and related disorders excluding severe psychotic”, “eating disorder”, etc.

Older age (age 40+) shows negative weight which is consistent with clinical presentations of BoPD occurring at younger ages^25^. When rating clinical records, our clinical expert did not consider gender as an important factor. And gender was not selected by the LR model trained on gold-label data either. Nonetheless, there was a large proportion of females with BoPD in the silver-positives data and this proportion was much greater in silver-positives (83%) than in silver-negative (63%), resulting a large positive weight in the learned LR model. We attribute this to the potential bias in clinical populations that a woman with BoPD may be more likely to seek treatment than a man with BoPD^26^. For this reason, we decided to include a gender feature in the model with the provision that it does not exclude the selection of males in the result.

While features such as anxiety and depression are common comorbid conditions of BoPD, they also commonly exist in other types of patients, including those with long term physical illness. As a result, the feature weights are not as large as expected. Similarly, non-compliance of medication is common for BoPD, and for other mental or physical disorders, and therefore this feature also does not have a large weight.

Furthermore, we also observe some features with effects in different directions from our clinical expert’s judgement, for example, “Agoraphobia with panic disorder” and “obsessive-compulsive disorder” were rated as negatively associated with BoPD, but their effects were positively associated in the LR model trained on silver-label data, potentially due to the fact that their prevalence is larger in silver-positive data than in silver-negative data. However, the prevalence of such features were generally small ($<5\%$) and we consider the impact of such misalignment with clinical expert’s judgement to be minimum on the prediction outcome. More discussion on features is in the Discussion section. Detailed feature information is in Supplementary Table 3.

## Methods

Here we introduce our proposed method for screening of BoPD, which follows a two-step approach by first selecting potential patients from general EHR data and then narrowing down the results of step 1 by applying ML prediction. The end product is a list of patients “most likely” to have BoPD that can be provided to health care professionals (HCPs) for further evaluation. We describe the selection rules for potential BoPD patients, data sets and feature engineering for ML development, and ML development pipeline in detail as follows. Refer to Supplementary Figure 1 for an illustration of the two-step approach.

### Cerner health facts database

For this study, the US-based Cerner Health Facts database was used to obtain the large sample of de-identified EHRs required for the algorithm development process. The database contains data extracted directly from electronic medical records provided by hospitals under a data use agreement with Cerner. Encounters may include pharmacy, clinical and microbiology laboratory, admission, and billing information from affiliated patient care locations. Date and time stamps are included for all admissions, medication orders and dispensing, laboratory orders and specimens, providing a temporal relationship between treatment patterns and clinical information. Cerner Corporation has established operating policies ensure that all data in the Health Facts database are fully de-identified in compliance with the Health Insurance Portability and Accountability Act. The data was collected from 2000 to 2018, with most encounters between 2009 and 2018.

### Selection rules for potential BoPD patients

**Table 2:** Mental disorder groups associated with BoPD and corresponding category code in Clinical Classifications Software Refined (CCSR) version 2020-02^27^.

Mental disorder groups associated with BoPD	CCSR category code
Depressive disorders	MBD002
Bipolar and related disorders	MBD003
Other specified and unspecified mood disorders	MBD004
Anxiety and fear-related disorders	MBD005
Trauma- and stressor-related disorders	MBD007
Disruptive, impulse-control and conduct disorders	MBD008
Personality disorders	MBD009
Suicidal ideation/attempt/intentional self-harm; initial and subsequent encounter	MBD012 and MBD027
Combined substance abuse related disorders including Alcohol-related disorders, Opioid-related disorders, Cannabis-related disorders, Sedative-related disorders, Stimulant-related disorders, Hallucinogen-related disorders, Inhalant-related disorders, and Other specified substance-related disorders	MBD017, MBD018, MBD019, MBD020, MBD021, MBD022, MBD023, and MBD025

We used the Clinical Classifications Software Refined (CCSR) version 2020.2^27^ to define the selection rules in a database queriable fashion as well as to construct features in the ML model. The CCSR aggregates over 70,000 ICD-10-CM codes into approximately 530 clinically meaningful categories. We considered default CCSR categories for each ICD-10-CM code in this study. Specifically, the selection rules for potential BoPD patients are :

Inclusion criteria:

- Patients had 5 or more encounters on different discharge dates or patients had 2 or more emergency visits on different discharge dates during study period;
- Patients ever had any diagnosis codes in the CCSR categories in Table 2 between the age of 18-65 years old during study period;
- Patients ever had suicidal/intentional self-harm (CCSR categories MBD012 and MBD027) or bipolar (CCSR category MBD003) during study period;
- Patients without suicidal/intentional self-harm (CCSR categories MBD012 and MBD027) or bipolar (CCSR category MBD003) must have had diagnosis codes in other mental disorders in at least 3 categories in Table 2 during study period;

Exclusion criteria:

- Patients who ever had BoPD diagnosis (ICD-9-CM of 301.83 or ICD-10-CM of F60.3) during the entire period of the EHR (if different from study period);
- Patients who ever had mental disorders due to known physiological conditions (ICD-10-CM codes F01-F09) or intellectual disabilities (ICD-10-CM codes F70-F79) during study period.

The clinical rationale of above selection criteria is described as follows. Bipolar Disorder and suicide symptoms are key indicators of the “likely BoPD” group (referred to as potential BoPD patients). The symptoms of BoPD and bipolar disorder overlap extensively^28,29^ and there is a high rate of misdiagnosis of BoPD as bipolar disorder^30^. Moreover, patients with BoPD have an increased risk of suicide^3^ which can be an indicator of affective instability and impaired emotion and behaviour regulation. Intentional self-harm (also referred to as non-suicidal self-injury (NSSI)) was included with suicidal behavior as they are in the same CCSR category (described in more details below) leading to easy data processing despite having different clinical presentations. The validity of these criteria is also supported by the observation that more than 70% of the EHR diagnosed BoPD cohort had either bipolar disorder or suicidal/intentional self-harm (Supplementary Table 1). For patients without Bipolar Disorder or suicidal symptoms including NSSI, the presence of other mental disorders in at least three pre-defined groups which are associated with BoPD was required for inclusion in the “likely BoPD” group. The intention was to capture a range of features of BoPD which may be reflected in individuals diagnosed with multiple disorders. We also aimed to avoid patients who primarily had a physical illness with secondary depression or anxiety.

In addition, unlike HCPs who are able to make clinical decision based on a single encounter with the patient, ML usually requires a larger amount of data to be trained and tested. We, therefore, required a minimum number of clinical encounters to ensure sufficient information for each patient to build the ML algorithm. Moreover, we suggest the study period to be the recent 4 to 5 years in practice, and therefore including time period when ICD-10-CM codes are in use.

### ML model development for screening BoPD

#### Data sets for ML model development

**Figure 5:**
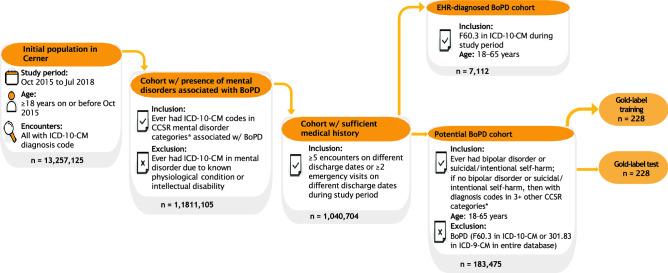
The flowchart of cohort selection using Cerner Health Facts data. $^*$Refer to Table 2 for CCSR mental disorder categories associated with BoPD.

The data sets to develop our ML algorithm consist of potential BoPD cohort, EHR diagnosed BoPD cohort and gold-label data sets based on Cerner data. We summarized the flowchart of obtaining the potential BoPD cohort, EHR diagnosed BoPD cohort and gold-label data sets in Cerner in Fig. 5. We considered encounters with ICD-10-CM diagnosis codes and a discharge date between October 1, 2015 and July 11, 2018 (last encounter discharge date in the Cerner database) to generate the data sets. Encounters without diagnosis codes were not considered. Applying the selection rules of potential BoPD patients to Cerner data in aforementioned study period yields the potential BoPD cohort. The EHR diagnosed BoPD cohort is defined as patients with EHR diagnoses of BoPD (ICD-10-CM code: F60.3) during study period. The additional inclusion criteria including age and minimum number of encounters, and exclusion criterion of known physiological conditions or intellectual disabilities are the same as criteria for the potential BoPD cohort. Gold-label training and testing data sets were two random samples drawn independently from the potential BoPD cohort, each with sample size 228 (456 in total). Our clinical expert provided ratings of the likelihood of having BoPD on these 456 patient records.

**Figure 6:**
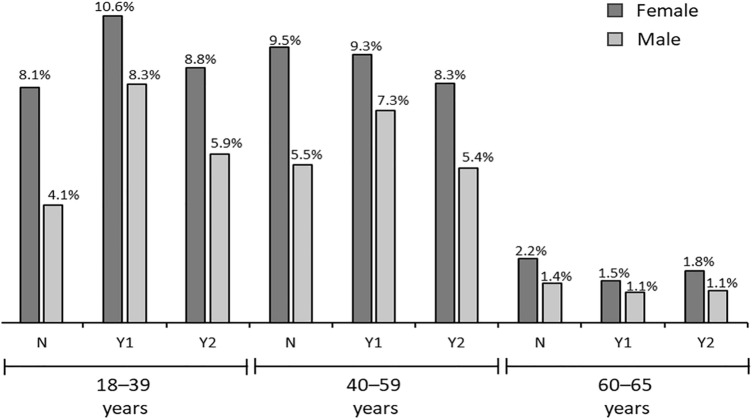
Stratification of potential BoPD cohort by age group, gender and diagnosis history on bipolar and suicidal or intentional self-harm behaviour. N: No diagnosis code of bipolar or suicidal/intentional self-harm and had $\ge 3$ categories in mental disorder categories in Table 2; Y1: Had diagnosis code of bipolar or suicidal/intentional self-harm and had $\ge 3$ categories in mental disorder categories in Table 2 (including bipolar or suicidal/intentional self-harm); Y2: Had diagnosis code of bipolar or suicidal/intentional self-harm but had $<3$ categories in mental disorder categories in Table 2 (including bipolar or suicidal/intentional self-harm).

Stratified sampling was used since it provides a better representation of the patient cohort as compared to simple random sampling. The strata were based on three variables: gender, age group and diagnostic history of bipolar and suicidal or intentional self-harm behaviour. Figure 6 shows detailed information for the description of each stratum and the corresponding prevalence in the potential BoPD cohort. We excluded 80 patients with ‘unknown or other’ gender from the random sampling.

**Figure 7:**
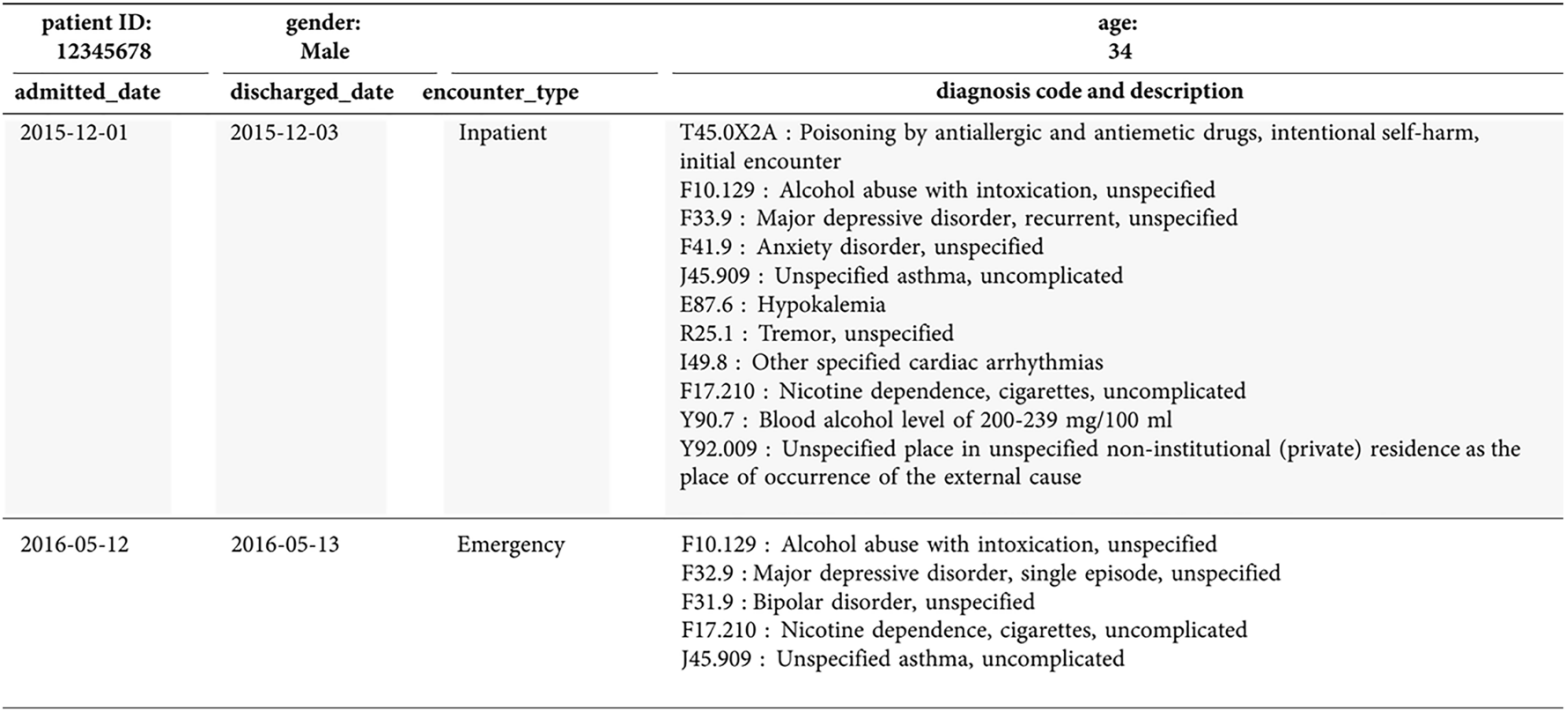
An illustration of a de-identified patient record presented to the clinical expert for rating.

For each patient in these two random samples, EHR data including gender, age, encounter types and dates, and diagnosis codes and the code descriptions associated with each encounter were presented to the clinical expert to be assigned to one of the mutually exclusive categories (category A-E) as shown in Table 3. Despite not having access to clinical notes, the available information was considered comprehensive enough to enable clinical judgement on the likelihood of a patient having BoPD. Pertinent information included diagnoses of suicide or self-harm, diagnoses that characterized both impulsivity and emotional instability (e.g. bipolar disorder and post-traumatic stress disorder), data implicating interpersonal difficulties (e.g. treatment non-compliance), a history of childhood trauma, or leaving the emergency room against medical advice. Patterns of treatment use were also considered, for example instability of outpatient care and multiple emergency room visits. The clinical expert reviewed patient records multiple times to ensure consistency of the ratings. The summary of our clinical expert’s ratings is shown in Table 3. A subset of patients in category “E: Most likely BoPD” were rated as having characteristics associated with classic BoPD (namely, category “E1: Classic BoPD”), which was defined as demonstrating diagnoses consistent with a minimum of 4 criteria of BoPD (e.g. suicide/self-injury, affective dysregulation, impulsivity, dissociation/psychosis under stress). We further showcased a snapshot of a de-identified patient record which was presented to the clinical expert in Fig. 7.

**Table 3:** Clinical expert’s rating in gold-label training and testing sets.

Rating category	Gold-label training set no. (%)	Gold-label testing set no. (%)
A: Not likely BoPD - primarily physical condition	37 (16%)	45 (20%)
B: Not likely BoPD - severe psychotic/substance abuse	28 (12%)	33 (14%)
C: Unsure - more information needed to make judgement	24 (11%)	19 (8%)
D: Possible BoPD	73 (32%)	70 (31%)
**E: Most likely BoPD**	**66 (29%)**	**61 (27%)**
- E1(a subset of E): Classic BoPD	12 (18%)	9 (15%)
Total	228 (100%)	228 (100%)

#### Feature engineering

We included a comprehensive list of covariates from our EHR data for predictive modeling, including: age (18-39, 40-59, 60-65), gender (Female/Male), categorization of encounters based on encounter frequency (high, median, low) and encounter types (emergency, inpatient, outpatient and others as one category), and diagnosis history. In particular, for diagnosis history, we first selected the top prevalent ($\ge 0.05$ ) ICD-10-CM diagnosis codes in both EHR diagnosed BoPD cohort and potential BoPD cohort, and then combined them to result in 571 ICD-10-CM diagnosis codes. Our clinical expert reviewed and rated these 571 diagnosis codes on their association with BoPD in the following categories: negative association, positive association, unrelated and unsure. We then reduced the dimension of the diagnosis codes to 220 by first mapping each diagnosis code onto their corresponding CCSR categories^27^. This ensured that the grouped codes share similar clinical meaning. Next, within each CCSR category, we further divided each mapped CCSR category by association with BoPD to include such knowledge into the model. Supplementary Figure 2 illustrates the method using diagnosis codes related to depressive disorder as an example. All features were encoded as Boolean value. Detailed definition of each feature can be found in the Supplementary Table 3.

#### ML development pipeline

Figure 1 illustrates the machine learning development pipeline to generate the screening model for step 2 (See the pseudo code in the Supplementary Algorithm 1). It follows a semi-supervised learning framework by starting with small gold-label data, and then building a large silver-label data. Model 1 was built on gold-label data as the initial binary classification model where category “E: Most likely BoPD” is the positive label, and other categories were combined to be negative labels. Model 1 was then applied to the unlabelled potential BoPD cohort (i.e. excluding gold-label sets) to generate predictions, and 14, 631 silver-negatives were randomly selected from those predicted as negative. The number of silver-negatives is determined by the number of silver-positives in order to have the same positive-to-negative ratio as in the gold-label training data (66 : 162). In the meanwhile, 5, 961 silver-positives were selected from the EHR diagnosed BoPD cohort following the same selection rules for potential BoPD cohort while excluding the BoPD diagnosis code, namely, patients had no bipolar nor suicidal/intentional self-harm and had 2 or less categories in mental disorder categories are excluded. Model 2 was then built on silver-label data as the main binary classification model for BoPD screening.

Because of the uniqueness of the rating category B (severe psychotic/substance abuse) as described in the Discussion section, Model 3 was built on a subset of the gold-label training data ($n=94$) which includes category “E: most likely BoPD” and “B: Not likely BoPD - severe psychotic/substance abuse” to distinguish these two categories. It was then combined with Model 2 in the following way to make adjustment to its prediction in order to reduce false positives, and therefore yielding the final model:

$$\begin{aligned} p_{\text {final}} = {\left\{ \begin{array}{ll} p_2, &{} \text {if }p_3 > 0.5\\ p_2 * p_3, &{} \text {otherwise}\\ \end{array}\right. } \end{aligned}$$

where $p_2$, $p_3$ and $p_{\text {final}}$ represent probability of positive generated by Model 2, Model 3 and final model, respectively. All models we used were logistic regression with L-1 norm regularization^31^, and in principal, any prediction model may be considered in this framework. In our case, regularized logistic regression shows its superiority in both prediction performance and interpretability and therefore was selected to be implemented in practice.

#### Model evaluation

The final performance of the developed model was evaluated on the (out-of-sample) testing data with gold labels. To prevent over-fitting, the best model was trained and selected on the training data by 10-fold cross-validation in terms of the area under the receiver operating characteristic (AUROC) on the validation set, and the testing data was kept unseen during model training procedure. We reported different classification metrics on the testing sets, including: AUROC and accuracy which need no threshold; and the positive predictive value and sensitivity at the threshold of 0.5 with specificity 0.922 (95% CI 0.880-0.960). We reported the 1,000-bootstrapped 95% confidence intervals for the above evaluation metrics^32^.

### Ethics

The Cerner Health Facts database is a real-world, de-identified, HIPAA-compliant electronic health records database. The study we conducted in this paper is on secondary use of this data and thus not considered as human subject research according to the definition of NIH (https://grants.nih.gov/policy/humansubjects/hs-decision.htm).

## Discussion

We investigated leveraging the use of EHR data and ML for screening patients who currently do not have BoPD diagnosis but may potentially have the disorder. The proposed approach was developed with the goal of providing an additional resource to facilitate clinical decision making and enabling scalable deployment of computational model in clinical practice. This approach is not meant to replace the clinical gold-standard screening (i.e. semi-structured interview with patients) nor to make a diagnostic decision, and HCPs should rely on their own judgement to make clinical decisions for an individual patient. Our results indicate that initial out-of-sample performance of ML model on the gold-label testing set is encouraging, showing high consistency with our clinical expert’s rating.

An important aspect of the proposed method is the two-step approach of initially rule-based selection, and then ML prediction to identify the candidate patients. The rule-based selection was used to improve the probability of identifying potentially under or misdiagnosed patients with BoPD, supported by nearly 30% patients rated as most likely BoPD in the gold-label sets. This not only creates relatively balanced training set for ML development and therefore avoids issues generated from imbalanced sample, it also made efficient use of clinical expert’s time to generate useful ratings on identifying most likely BoPD patients from potential patients instead of from general subjects. The rule-based selection excludes subjects either for not having common comorbidities or key characteristics of BoPD, or not having sufficient diagnosis history for ML prediction. The clinical rationale of selection rules is explained in the Methods section.

In the ML development, the construction of a larger silver-label data may improve generalizability in real-world practice with initial demonstration described in the Result section. Silver-negatives can only be selected with guidance from gold-label training data, and silver-positives, on the other hand, can be built in multiple ways. We chose to use EHR diagnosed BoPD cohort instead of potential BoPD cohort, because EHR diagnosed BoPD cohort represents a collection of clinical expert opinions which is more diversified from our clinical expert’s judgement reflected in the gold-label data. We chose to use rule-based selection instead of gold-label guided selection, because the selection rules are based on common comorbidities and key characteristics of BoPD, and it potentially introduces additional information different from knowledge learned from gold-label guided selection, and therefore may improve generalizability performance of the developed ML model.

The comparison of the coefficients of LR models built on gold-label data and silver-label data (blue and red bars in Fig. 4) reveals that, clinical highly relevant features stand out more in the model trained on silver-label data when they are not selected by model trained on gold-label data, such as unspecified personality disorder and bipolar disorder. This further supports the strategy of building silver-label data instead of only using gold-label data.

We noticed that several features such as Schizophrenia and Hallucinations were only highly prevalent in patients rated as “severe psychotic/substance abuse” which is a subset of the negative labels. However, these features did not stand out in the main model, because their effects have been diluted when “severe psychotic/substance abuse” was combined with other rating categories as negative labels. Therefore, the main model may wrongly classify such patients as positive cases. To bring out such feature effects and to reduce false positive, we have built an adjustment model on a subset of the gold-label training set with ratings only in “severe psychotic/substance abuse” or “most likely BoPD” to distinguish the two. Such features indeed showed up to be important in the adjustment model (green bars in Fig. 4). The benefit of including adjustment model seems to be marginal in the overall model performance (Fig. 2), and it may be improved by changing the rule when combining with the main model, and/or improving the adjustment model itself by expanding the training set analogous to the construction of silver label data for the main model.

We have considered formulating the problem as multi-class classification instead of binary classification, however, the goal is to identify “most likely BoPD” from others which is a natural binary consideration, and due to limited sample size of gold-label data, multi-class classification is not a promising modeling direction.

This study has several limitations. The observation that not all patients from EHR diagnosed BoPD cohort were not included in silver-positives due to limited comorbidities in the structured EHR reflect a limitation of our EHR data. Our data is from a single EHR provider, while patients may have encounters with different providers in different facilities using different EHR systems, raising the potential for missing data. Nevertheless, medical records may not be easily transferred between different systems and HCPs may only have access to the local EHR; thus, use of a single EHR may be more representative of the ‘real-world’ situation. Our primary modeling features are ICD-10-CM codes which are US-specific, and may not be directly applicable to other countries where other ICD codes are used. Additionally, medical notes were not available to us and therefore were not included within the analyses which limits the available clinical information. Moreover, there was only one clinical expert to provide gold-labels, although we tried to include diversified clinical expert opinions when constructing the silver-label data, as discussed previously.

When developing the screening algorithm, model interpretability was important because providing clarity and transparency into how the algorithm makes decision is critical to us. In fact, we have explored several machine learning models including “black-box” models, and the interpretable model stands out. Scalability was also an important factor that we considered in the design of the proposed method. We grouped the diagnosis codes to potentially overcome heterogeneity in coding behavior among HCPs. In addition, we opted for simple structured EHR data which are routinely collected in all EHR systems with standardized coding, although is a limitation of the current method, but may increase the chance for scalable deployment.

While the ML model achieved good performance on the out-of-sample gold-label testing data, and demonstrated high consistency with our clinical expert’s ratings, we were not able to confirm whether the patients identified as “most likely BoPD” are eventually diagnosed with BoPD. This would require semi-structured interviews on patients from the Cerner database which was not possible with the de-identified data that was supplied to us. When we applied the model to silver-positives which includes patients with a BoPD diagnosis code, we found that 62% of the silver-positives were predicted as “most likely BoPD” by the model. This result is from an in-sample evaluation and may not be generalizable. Nonetheless, our ML model shows a 72% positive predictive value and 100% recall of the “classic BoPD” sub-category in the out-of-sample performance. Overall, we think the proposed method is a potentially helpful strategy to identify possible BoPD patients who are otherwise undiagnosed.

Application of the BoPD screening tool in a real-world setting is currently underway but does require consideration of several factors. The implementation requires access to EHR data at sites and the ability to pre-process the data with pre-determined logic and format, which may only be feasible for research-oriented health institutes. Due to data privacy concerns, we are not able to access the site data and produce results for end users; however, we have made the code available on GitHub with a user-friendly interface and have written a detailed user manual with sample SQL codes to help sites understand the data pre-processing logic in order to facilitate adoption. Intermediaries such as an EHR data aggregator or EHR system provider may be a good candidate to adopt our model in their platform, alleviating analytical burden for sites. In addition, there may be operational challenges along the way such as getting model results and patient re-identification to end users in a seamless fashion. Last but not least, end users need to follow their IRB regulations for re-identification of screened patients and subsequent contact with patients.

In conclusion, we developed a machine learning-based screening model to identify possible BoPD patients who are otherwise undiagnosed using structured EHR data. Our model integrated both clinical rule-based selection and a semi-supervised learning framework, leading to better generalization performance than models learned from gold-labeled data. Our study suggested the promise of developing scalable computational tools for BoPD screening and diagnosis based on machine learning and EHR data.

## Supplementary Information

Supplementary Information 1.

Supplementary Information 2.

Supplementary Information 3.

## Data Availability

The data sets analyzed during the current study are not publicly available due to restrictions by Cerner; the owner of the data. Data could be accessed by signing a data sharing agreement with Cerner and covering any costs that may be involved.

## Code availability

The BoPD screening algorithm has been integrated into a screening tool for implementation. There are two versions: a portable version which has a graphical interface and a headless version which includes executable Python source code without a graphical interface. In addition, instruction and sample SQL code for EHR data processing are included in a user manual. All are publicly available at https://github.com/BoPDdiseasescreening/Borderline-Personality-Disorder-BoPD-automatic-disease-screening-tool. The portable version of the screening tool is demonstrated in https://github.com/BoPDdiseasescreening/Borderline-Personality-Disorder-BoPD-automatic-disease-screening-tool/blob/main/BoBP_screening_portableversiondemo.mp4.
