# Supplementary material for: Development of a screening algorithm for borderline personality disorder using electronic health records
Source: Sci Rep. 2022 Jul 13;12:11976. doi: 10.1038/s41598-022-16160-z (PMC9279396; doi:10.1038/s41598-022-16160-z)
Supplement: Supplementary file 1 — Supplementary Information 1. [file 41598_2022_16160_MOESM1_ESM.pdf]

Supplemental Materials for
Development of a screening algorithm for borderline personality disorder using electronic health records

Chengxi Zang, Marianne Goodman, Zheng Zhu, Lulu Yang, Ziwei Yin, Zsuzsanna Tamas, Vikas Mohan Sharma, Fei Wang, Nan Shao

- Supplementary Figure 1: Overall screening algorithm follows a two-step approach by first selecting potential patients from general EHR data and then narrowing down the results of step 1 by applying ML prediction. The end product is a list of patients "most likely" to have BoPD that can be provided to HCPs for further evaluation.
- Supplementary Figure 2: An example of grouping depressive disorder-related diagnosis codes in feature engineering. Clinical knowledge of positive or negative association of diagnosis codes with BoPD were explicitly encoded.
- Supplementary Table 1: Patient demographics and encounter characterization of potential BoPD cohort and EHR diagnosed BoPD cohort.
- Supplementary Table 2 [File attached]: Model performance metrics of different machine learning models trained on different data sets.
- Supplementary Table 3 [File attached]: Features included in the model training process, their description, coefficients in different models and prevalence in both the training and test data sets.
- Supplementary Algorithm 1: The semi-supervised framework of our machine learning model for screening BoPD patients.

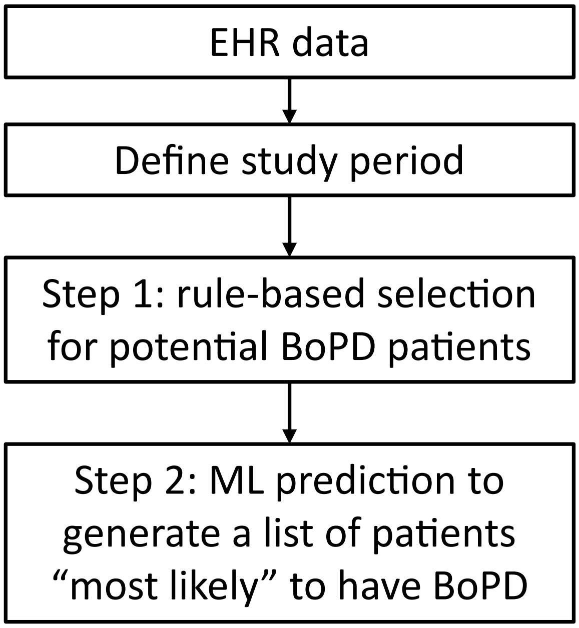

Supplementary Fig. 1. Overall screening algorithm follows a two-step approach by first selecting potential patients from general EHR data and then narrowing down the results of step 1 by applying ML prediction. The end product is a list of patients “most likely” to have BoPD that can be provided to HCPs for further evaluation.

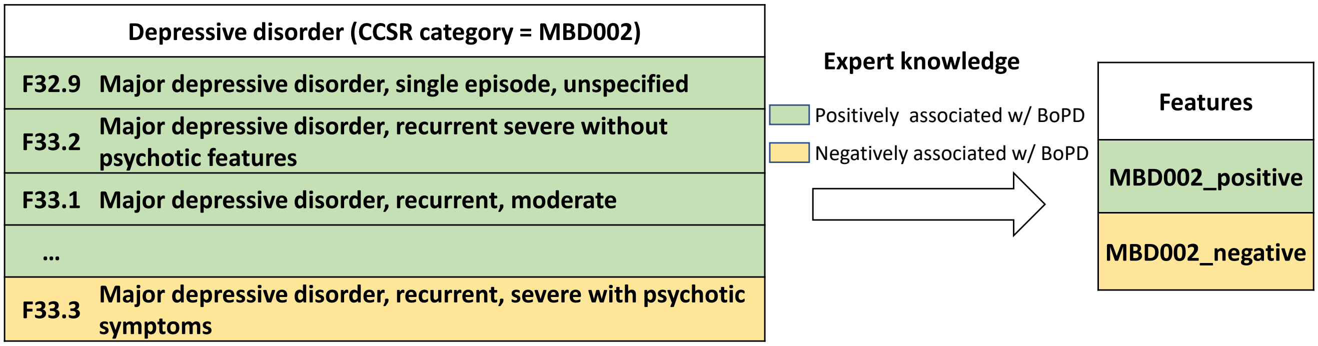

Supplementary Fig. 2. An example of grouping depressive disorder-related diagnosis codes in feature engineering. Clinical knowledge of positive or negative association of diagnosis codes with BoPD were explicitly encoded.

Supplementary Table 1. Patient demographics and encounter characterization of potential BoPD cohort and EHR diagnosed BoPD cohort.

	Potential BoPD cohort	EHR diagnosed BoPD cohort
Number of subjects	183,475	7,112
Age group, no. (%)		
18–39 years	83,942 (45.7%)	4,360 (61.3%)
40–59 years	83,042 (45.3%)	2,435 (34.2%)
60–65 years	16,491 (9.0%)	317 (4.5%)
Gender, no. (%)		
Female	110,284 (60.1%)	5,879 (82.7%)
Male	73,151(39.9%)	1,227 (17.3%)
Unknown or other	40 (<0.1)	6 (<0.1)
Duration between the first encounter and the last encounter, days		
Mean (SD)	525.2 (282.5)	544.9 (286.2)
Median (range)	536 (2–1,004)	553.5 (2–1,004)
Number of encounters per subject		
Mean (SD)	14.6 (16.1)	20.3 (22.8)
Median (range)	9 (2–419)	12 (2–246)
Encounter type, no. of encounters (%)		
Emergency	717,289 (25.8%)	31,349 (23.2%)
Inpatient	177,159 (6.4%)	9,484 (7.0%)
Outpatient and other*	1,881,071 (67.8%)	94,049 (69.7%)
Comorbidity **, no. (%)		
Y1	69,727 (38.0%)	4,405 (62.0%)
Y2	57,275 (31.2%)	587 (8.2%)
N	56,473 (30.8%)	969 (13.6%)
None of above	0 (0%)	1,151 (16.2%)

* Majority of the "other" encounter type included clinic, recurring, not mapped, other specialty, observation.

** The definition of Y1, Y2 and N is the same as in the stratification figure. For EHR diagnosed BoPD cohort, BoPD diagnosis code was not used in the comorbidity analysis.

N: No diagnosis of bipolar or suicidal/intentional self-harm

Y1: Patient diagnosed as bipolar or suicidal/intentional self-harm and have ≥3 categories in mental disorder categories in CCSR table (including bipolar or suicidal/intentional self-harm)

Y2: Patient diagnosed as bipolar or suicidal/intentional self-harm but have <3 categories in mental disorder categories in CCSR table (including bipolar or suicidal/intentional self-harm)

Supplementary Algorithm 1 The semi-supervised framework of our machine learning model for screening BoPD patients

Input:
(X_{Gold}, Y_{Gold}): the small gold-labeled potential BoPD patients data
$X_{\text{Unlabeled}}$: the large unlabeled potential BoPD patients data,
X_{DX}: the diagnosed BoPD patients data;
$\mathscr{F}_{\Theta}$: a set of machine learning binary classification models;
Output: f_{final}: the final output model

1: training and selecting the gold model $f_{\theta}^{(1)} \in \mathscr{F}_{\Theta}$ using the small gold-labeled data (X_{Gold}, Y_{Gold});
2: building silver negative data ($X_{\text{Silver-}}$, -1) by applying $f_{\theta}^{(1)}$ to $X_{\text{Unlabeled}}$ and selecting n_{s-} data samples with the top predicted negative labels;
3: building silver positive data ($X_{\text{Silver+}}$, +1) by randomly selecting n_{s+} from X_{DX}
4: building silver-labeled data (X_{Silver}, Y_{Silver}) by combining ($X_{\text{Silver+}}$, +1) and ($X_{\text{Silver-}}$, -1) with the ratio $\frac{n_{s+}}{n_{s-}}$ the same as the gold-labeled data
5: training and selecting the silver model $f_{\theta}^{(2)} \in \mathscr{F}_{\Theta}$ using the silver-labeled data (X_{Silver}, Y_{Silver});
6: training and selecting the adjustment model $f_{\theta}^{(3)} \in \mathscr{F}_{\Theta}$ using the gold-labeled data (X_{Gold}, Y_{Gold});
7: building the final model f_{final} by ensembling both the silver model $f_{\theta}^{(2)}$ and the adjustment model $f_{\theta}^{(3)}$
8: **return** f_{final}
